# Comparative Study of Cu Ion Adsorption by Nano-Hydroxyapatite Powder Synthesized from Chemical Reagents and Clam Shell-Derived Calcium Sources

**DOI:** 10.3390/nano14171431

**Published:** 2024-09-01

**Authors:** Shih-Ching Wu, Hsueh-Chuan Hsu, Hong-Yi Ji, Wen-Fu Ho

**Affiliations:** 1 Department of Dental Technology and Materials Science, Central Taiwan University of Science and Technology, Taichung 406053, Taiwan; scwu@ctust.edu.tw (S.-C.W.); hchsu@ctust.edu.tw (H.-C.H.); 2 Department of Chemical and Materials Engineering, National University of Kaohsiung, Kaohsiung 811726, Taiwan

**Keywords:** clam shells, Cu ions, heavy metal adsorption, hydroxyapatite, microwave synthesis

## Abstract

The increasing contamination of water sources by heavy metals necessitates the development of efficient and sustainable adsorption materials. This study evaluates the potential of nano-hydroxyapatite (HA) powders synthesized from chemical reagents (Chem-HA) and clam shells (Bio-HA) as adsorbents for Cu ions in aqueous solutions. Both powders were synthesized using microwave irradiation at 700 W for 5 min, resulting in nano-sized rod-like particles confirmed as HA by X-ray diffraction (XRD). Bio-HA exhibited higher crystallinity (67.5%) compared to Chem-HA (34.9%), which contributed to Bio-HA’s superior adsorption performance. The maximum adsorption capacities were 436.8 mg/g for Bio-HA and 426.7 mg/g for Chem-HA, as determined by the Langmuir isotherm model. Kinetic studies showed that the Cu ion adsorption followed the pseudo-second-order model, with Bio-HA achieving equilibrium faster and displaying a higher rate constant (6.39 × 10⁻^4^ g/mg·min) than Chem-HA (5.16 × 10⁻^4^ g/mg·min). Thermodynamic analysis indicated that the adsorption process was spontaneous and endothermic, with Bio-HA requiring less energy (ΔH° = 39.00 kJ/mol) compared to Chem-HA (ΔH° = 43.77 kJ/mol). Additionally, the activation energy for Bio-HA was lower (41.62 kJ/mol) than that for Chem-HA (46.39 kJ/mol), suggesting better energy efficiency. The formation of a new Cu_2_(OH)PO_4_ phase after adsorption, as evidenced by XRD, confirmed that the Cu ions replaced the Ca ions in the HA lattice. These findings demonstrate that Bio-HA, derived from natural sources, offers environmental benefits as a recyclable material, enhancing heavy metal removal efficiency while contributing to sustainability by utilizing waste materials and reducing an environmental impact.

## 1. Introduction

With the development of urbanization and industrialization, heavy metal pollution has become a serious environmental issue. The major sources of metal pollution typically stem from industrial wastewater, mining, smelting, electroplating, and discarded batteries [1,2]. Heavy metal pollutants are often present in liquid form, and when humans are exposed to these metals, they cannot be metabolized and excreted, leading to accumulation of metal concentrations in the body. When the levels exceed the standard limits, it can result in various diseases. Common metal waste liquids include Cu^2+^, Zn^2+^, Cr^2+^, and Ni^2+^. Treatment methods for these pollutants include chemical precipitation, solvent extraction, electrolytic processes, reverse osmosis, chemical oxidation or reduction, and adsorption [3,4,5,6,7]. Among these methods, adsorption stands out due to its low cost, low energy consumption, simplicity, recyclability, and high efficiency [8,9]. Common adsorbents include activated carbon, zeolites, and biochar [10,11]. Recent studies have focused on using biological waste to prepare adsorbents, such as sugarcane bagasse, bovine bones, and fish bones [11,12,13,14]. This approach not only reduces the cost of adsorbents but also promotes the circular economy by reusing waste materials.

Hydroxyapatite (HA) is the primary inorganic component of bones and teeth [15,16], widely used as a bone implant material, a drug delivery system, photocatalyst, and an adsorbent. Due to its non-toxic nature to both biological systems and the environment, HA adsorbents can be applied in environmental pollution treatment, such as soil contamination and industrial wastewater [17]. HA possesses a high surface area, high stability, low solubility, and excellent adsorption properties [16,18]. The functional group Ca–OH can undergo ion exchange with metal ions in waste liquids, while the O–H functional group facilitates the adsorption of metal ions onto the surface of HA [19]. HA is particularly effective in adsorbing divalent metals, with its Ca^2+^ ions capable of ion exchange with divalent metal ions, making it a promising adsorbent for metals such as Cu^2+^, Pb^2+^, Ni^2+^, and Cr^2+^. Research by Hernández-Cocoletzi et al. [20] demonstrated that HA is highly effective in adsorbing divalent metals like Cu^2+^, Ni^2+^, and Zn^2+^, achieving removal rates of over 95%. There are documented cases of using biomass waste, such as fish bones [13] and bovine bones [12], to produce HA for the adsorption of metal ions [16] and organic compounds [21] from industrial wastewater. Cu is a common metal in both industrial and domestic applications, frequently used in electroplating plants, fastener manufacturers, and chemical factories. In particular, waste liquid from circuit board manufacturing contains high concentrations of Cu. If Cu and Cu^2+^ solutions used in factories are not properly treated and are released into the environment, they can contaminate water sources and come into contact with humans. Ingesting more than 10 mg of Cu ions can cause nausea, vomiting, and abdominal pain, while long-term exposure can lead to liver diseases [22]. Therefore, reducing the environmental and human health risks posed by Cu^2+^ is an urgent and critical issue. The study by Yang et al. [23] demonstrated that the Langmuir model best describes the equilibrium data, with the estimated maximum adsorption capacity of poorly crystallized HA adsorbent for Cu ions reaching 41.80 mg/g at 313 K. Ayodele et al. [24] investigated the adsorption of Cu ions from both synthetic and industrial wastewater on HA derived from eggshells. The maximum adsorption capacity of HA for Cu ions was 10.58 mg/g. Moreover, HA could be reused, and Cu ions could be recovered. Additionally, HA-based adsorbents have been widely studied. Yang et al. [25] also reported that at 313 K, humic acid-modified HA nanoparticles had an estimated maximum adsorption capacity of 58.42 mg/g. Therefore, HA is suitable as an effective adsorbent material for the removal of Cu ions from aqueous solutions.

Clams are commonly consumed seafood, and their shells, which are often discarded as waste, lack stable and proper disposal methods. The primary component of clam shells is calcium carbonate, which can be used as a Ca source for synthesizing HA. There are several methods for synthesizing HA, with the most common ones being the sol–gel method [22], the hydrothermal method [26,27], and the precipitation method [28,29]. However, these methods are time-consuming, and the hydrothermal method is also energy-intensive. In this study, the microwave method was chosen for its simplicity, short processing time, low energy consumption, and the ability to produce molecules with high uniformity [30,31], making it more advantageous compared to traditional synthesis methods.

To the best of the authors’ knowledge, there has been no literature comparing the adsorption effects and characteristics of HA synthesized from chemical reagents and biomass waste, making it difficult to compare the benefits of each. Therefore, this study aims to synthesize HA using chemical reagent calcium oxide and clam shells, producing Chem-HA and Bio-HA, respectively. This is the first study to compare the adsorption characteristics of HA synthesized from chemical reagents and biomass materials. In this research, Chem-HA and Bio-HA powders are synthesized by controlling the microwave power at 700 W and a reaction time of 5 min. After the adsorption of Cu ions, the solution is analyzed using UV-VIS to evaluate the adsorption effectiveness. Additionally, adsorption kinetics and thermodynamics calculations are performed to explore the adsorption mechanisms and efficiency of Chem-HA and Bio-HA.

## 2. Materials and Methods

### 2.1. Preparation of Chem-HA and Bio-HA Adsorbents

In this study, two sources of Ca were used to prepare HA powders under the same process conditions. The HA powder synthesized from chemical reagent is labeled as Chem-HA, while the HA powder synthesized from clam shell powder is labeled as Bio-HA. To prepare the adsorbents, 1.27 g of clam shell powder and 0.71 g of CaO were each added to 25% acetic acid solutions, respectively. The mixtures were stirred using a magnetic stirrer for 3 h to ensure the complete dissolution of the powders, resulting in Ca source solutions. Separately, 1.0 g of diammonium hydrogen phosphate was dissolved in 3 mL of water, and this phosphate solution was added to the Ca solutions. The pH was adjusted to 10 using an ammonia solution. The prepared solutions were then placed in a household microwave oven (Panasonic, NN-SD681, Shanghai, China) and reacted at power of 700 W for 5 min. After the microwave treatment, the solutions underwent centrifugation and ultrasonic agitation, which was repeated five times to complete the washing process. Finally, the washed precipitates were dried in an oven at 100 °C for 24 h to obtain the Chem-HA and Bio-HA powders.

### 2.2. Preparation of a Cu Ion Solution

A solution containing Cu ions was prepared by dissolving 3.929 g of copper (II) sulfate pentahydrate in 1000 mL of deionized (DI) water. The mixture was stirred with a glass rod until the copper (II) sulfate pentahydrate was completely dissociated in the DI water, resulting in a Cu ion solution.

### 2.3. UV-VIS Spectroscopy Analysis

Zengin et al. [32] used UV-VIS spectroscopy (Cintra 3030, GBC Scientific Equipment, Keysborough, Australia) to measure the concentration of Cu ions in a solution, determining the remaining Cu ion concentration by analyzing the absorbance spectra. Their results, corroborated by subsequent ICP analysis, showed an error margin of approximately 3.3–5% between the UV-VIS and ICP methods. Based on this, UV-VIS was employed in this study for the analysis of Cu ion concentration. First, Cu ion solutions of various concentrations (100, 200, 300, 400, 500, 600, 700, 800, 900, and 1000 mg/L) were prepared. UV-VIS spectroscopy was used to analyze the absorbance spectra of these solutions, generating standard spectra for each concentration. Solutions containing Cu ions, after adsorption by Chem-HA and Bio-HA materials, were measured using UV-VIS spectroscopy. The observation wavelength was set between 600 and 900 nm, and the peak value at 810 nm [33] was used for comparison with the standard spectra. The peak values obtained were interpolated to calculate the remaining Cu ion concentration in the solution and the amount of Cu ions adsorbed by the materials. The following formulas were used for calculations [34]:

(1) $$\mathrm{Removal}\;(\%)=\frac{C_{0}-C_{e}}{C_{0}}\times 100\%$$

(2) $$\mathrm{Adsorption}\;\mathrm{capacity}\;(q_{e})=\frac{{(C}_{0}-C_{e})V}{W}$$

where C_0_ (mg/L) is the initial concentration of Cu ions in the solution, C_e_ (mg/L) is the equilibrium concentration of Cu ions in the solution, q_e_ (mg/g) is the amount of Cu ions adsorbed on the adsorbent at equilibrium, V (L) is the volume of the solution, and W (g) is the mass of the adsorbent.

### 2.4. Structural and Morphological Observation

The Chem-HA and Bio-HA powders synthesized by the microwave method were subjected to Cu ion adsorption experiments. Before and after the adsorption experiments, the powders were ground using an agate mortar and analyzed by X-ray diffraction (XRD; D8 Advance, Bruker, Berlin, Germany). The XRD analysis utilized a copper target with a wavelength of 0.15406 nm, operating at a voltage of 30 kV and a current of 30 mA. The scanning speed was set at 2°/min, with a diffraction angle (2θ) range of 20° to 50°.

The surface of the powders before and after Cu ion adsorption experiments was coated with platinum (current of 10 mA, sputtering time of 180 s). The morphology of Chem-HA and Bio-HA was observed using a field emission scanning electron microscope (FE-SEM; S-4800, Hitachi, Tokyo, Japan).

### 2.5. Adsorption Kinetics

Adsorption kinetics are primarily used to evaluate the efficiency and mechanism of the adsorption process. The kinetics of the adsorption process are mainly interpreted through two kinetic models: the pseudo-first-order model and the pseudo-second-order model. The equations are as follows [35]:

Pseudo-first-order model: ln (q_e_ − q_t_) = ln q_e_ − k_1_t (3)

(4) $$\mathrm{Pseudo-}\mathrm{second-}\mathrm{order}\;\mathrm{model}\text{:}\;\frac{t}{q_{t}}=\frac{1}{k_{2}{q_{e}}^{2}}+\frac{t}{q_{e}}$$

where q_e_ (mg/g) is the amount of Cu ions adsorbed on the adsorbent at equilibrium, q_t_ (mg/g) is the amount of Cu ions adsorbed on the adsorbent at time t (min), k_1_ (1/min) is the pseudo-first-order adsorption rate constant for Chem-HA and Bio-HA, and k_2_ (g/mg·min) is the pseudo-second-order adsorption rate constant for Chem-HA and Bio-HA.

The pseudo-first-order model assumes that the difference between the adsorption capacity at equilibrium and at time t is based on a fixed concentration, and it is obtained by plotting ln (q_e_ − q_t_) against time t. The pseudo-second-order model assumes that chemical adsorption occurs between the adsorbent and the adsorbate, and it is obtained by plotting t/q_t_ against time t.

In this experiment, the solution temperature was set at 27 °C, the initial concentration of the Cu ion solution was 200 mg/g, the volume of the Cu ion solution was 20 mL, and the amounts of Chem-HA and Bio-HA added were 0.05 g. The contact time ranged from 5 to 240 min. The kinetic calculations provided insights into the reaction rates of Chem-HA and Bio-HA adsorptions and the changes between the adsorbent and the adsorbate.

### 2.6. Adsorption Isotherms

Adsorption isotherms describe the equilibrium of a substance adsorbed on a surface at a given temperature. They indicate the amount of the substance bound to the surface as a function of its concentration in the solution. The adsorption behaviors of Chem-HA and Bio-HA were studied using two commonly used isotherms: Langmuir and Freundlich [12].

(5) $$\mathrm{Langmuir}\;\mathrm{model}\text{:}\;\frac{C_{e}}{q_{e}}=\frac{1}{K_{L}q_{m}}+\frac{C_{e}}{q_{m}}$$

(6) $$\mathrm{Freundlich}\;\mathrm{model}\text{:}\;lnq_{e}=lnK_{F}+\left(\frac{1}{n}\right)lnC_{e}$$

where C_e_ (mg/L) is the concentration of Cu ions in the solution at equilibrium, q_e_ (mg/g) is the amount of Cu ions adsorbed on the adsorbent at equilibrium, K_L_ (L/mg) is the Langmuir adsorption constant, K_F_ (L^1/n^·mg^(1−1/n)^·g^−1^) is the Freundlich adsorption constant, q_m_ (mg/g) is the maximum monolayer adsorption capacity, and n (g/L) is the adsorption intensity.

The Langmuir model assumes that the adsorption process occurs on a homogeneous surface with a monolayer adsorption pattern, whereas the Freundlich model assumes a heterogeneous surface with multilayer adsorption. In this experiment, the adsorption behaviors of Chem-HA and Bio-HA adsorbents were studied at a solution temperature of 27 °C with initial Cu ion concentrations of 500, 600, 700, 800, 900, and 1000 mg/L. An adsorbent amount of 0.02 g was used. After a contact time of 24 h, UV-VIS analysis was performed. By comparing the Langmuir and Freundlich models, the mechanisms between the adsorbent and the adsorbate can be understood, and the maximum adsorption capacity under ideal conditions can be determined.

### 2.7. Adsorption Thermodynamics

Thermodynamic experiments were conducted to understand the effect of solution temperature on the adsorption process, providing a clearer understanding of the adsorption mechanism. The equations used are as follows [12]:

∆G = ∆H° − T ∆S°(7)

K_0_ = q_e_/C_e_ (8)

ln K_0_ = ∆S°/R − ∆H°/RT (9)

where ΔG (J/mol) is the Gibbs free energy change for Chem-HA and Bio-HA, R is the gas constant (8.314 J/K·mol), T (K) is the experimental solution temperature, K_0_ (L/g) is the adsorption thermodynamic equilibrium variable, ∆S° (J/mol·K) is the entropy change for Chem-HA and Bio-HA, and ∆H° (J/mol) is the enthalpy change for Chem-HA and Bio-HA.

In this study, four different solution temperatures (308, 313, 318, and 323 K) were used to assess the adsorption capacity of Chem-HA and Bio-HA at an initial Cu ion solution concentration of 500 mg/L. Using the equations above, the ΔG for Chem-HA and Bio-HA at different temperatures were calculated. From the ΔG obtained, the ∆H° and ∆S° for Chem-HA and Bio-HA were further calculated to understand the exothermic and endothermic nature of the overall adsorption process. The results indicate whether the adsorption process is spontaneous or non-spontaneous.

## 3. Results and Discussion

### 3.1. Structural and Morphological Analysis of Chem-HA and Bio-HA Powders before and after Cu Ion Adsorption

**Figure 1:**
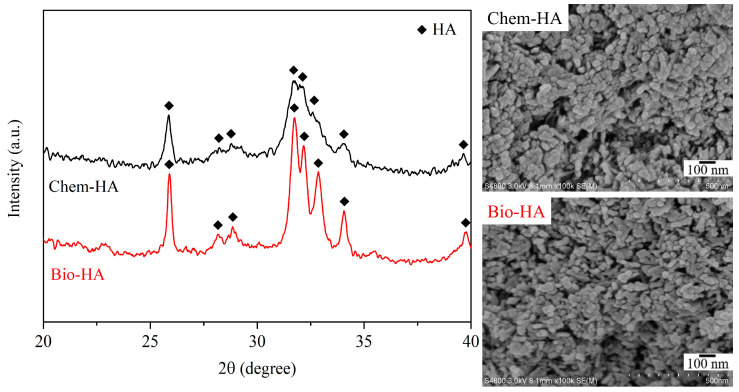
XRD patterns and SEM images of Chem-HA and Bio-HA powders prepared using chemical calcium oxide and clam shells, respectively.

Figure 1 presents the XRD patterns and SEM images of the synthesized Chem-HA and Bio-HA powders. The XRD results confirm that both powders are composed of HA (JCPDS file No. 09-0432), with Bio-HA showing sharper diffraction peaks compared to Chem-HA, indicating higher crystallinity. The crystallinity values for Chem-HA and Bio-HA powders are 34.9% and 67.5%, respectively. The SEM images reveal that both powders consist of nano-sized rod-like particles with rough surfaces. Based on SEM analysis, the average particle sizes of the Chem-HA and Bio-HA powders were approximately 25.5 nm and 37.5 nm, respectively. Additionally, BET analysis revealed that the specific surface areas of Chem-HA and Bio-HA were 80.0 m^2^/g and 61.9 m^2^/g, respectively. The difference in crystallinity and particle size between Chem-HA and Bio-HA can be attributed to the distinct sources of Ca used in their synthesis. Chem-HA, synthesized from chemical reagents, may exhibit lower crystallinity due to the controlled and pure nature of the reagents, which results in less impurity incorporation. In contrast, Bio-HA, synthesized from clam shell powder, contains more impurities, such as Na and Mg, which are naturally present in clam shells. These impurities can influence both crystallinity and particle size. Specifically, they can act as nucleation sites, potentially leading to variations in crystal growth and, in some cases, promoting higher crystallinity. Additionally, the presence of these impurities may contribute to an increase in particle size, as seen in Bio-HA, where the average particle size is larger compared to that of Chem-HA. This variation highlights how starting materials affect both the structural properties and particle characteristics of HA.

**Figure 2:**
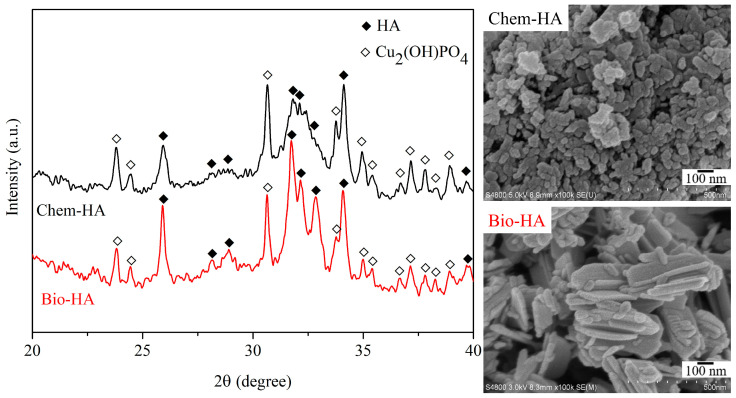
XRD patterns and SEM images of Chem-HA and Bio-HA powders after Cu ion adsorption.

Figure 2 shows the XRD patterns and SEM images of Chem-HA and Bio-HA powders after Cu ion adsorption. The XRD patterns indicate the formation of a new Cu_2_(OH)PO_4_ phase (JCPDS file No. 36-0404), demonstrating that Cu^2+^ ions replaced Ca^2+^ ions in the HA lattice, resulting in a new crystalline phase. After Cu ion adsorption, the crystallinity of Chem-HA and Bio-HA decreased to 12.3% and 60.7%, respectively. This reduction in crystallinity is consistent with the findings by Othmani et al. [34], who reported a similar decrease when Cu ions substituted Ca ions in HA. SEM images show an increase in particle size for Chem-HA and Bio-HA powders after Cu ion adsorption, with Chem-HA particles becoming more spherical due to the incorporation of Cu ions into the HA lattice. Othmani et al. [36] noted that copper ions cause the HA crystal to contract along either the c-axis or the a-axis.

### 3.2. Adsorption Kinetics Models

**Figure 3:**
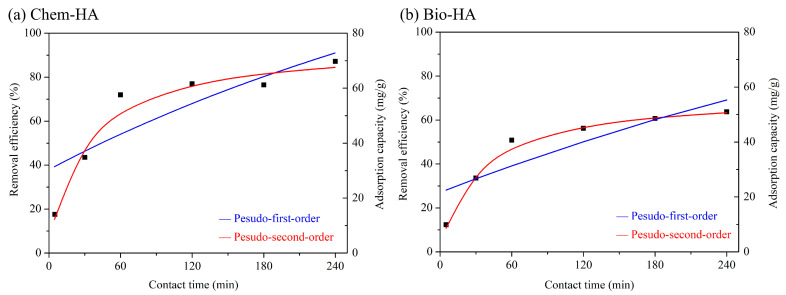
Removal efficiency and adsorption capacity of Cu ions from a 200 mg/L solution by (**a**) Chem-HA and (**b**) Bio-HA as adsorbents at various contact times.

Kinetic studies are crucial for determining the reaction constants of adsorbent materials and understanding how solution concentration affects the contact time with the adsorbent, thereby revealing the efficiency of the adsorbent at specific contact times. In preliminary tests, UV-VIS analysis showed minimal changes in adsorption at high Cu ion concentrations, making it challenging to calculate the adsorption capacity. Therefore, experiments were conducted with a lower Cu ion concentration (200 mg/L) and a higher adsorbent dose (0.05 g). The results from the kinetic equations indicate that the adsorption behavior of both Chem-HA and Bio-HA follows a pseudo-second-order model, with correlation coefficients of 0.989 and 0.998, respectively [24]. During the initial 60 min of the adsorption process, Cu ions quickly adsorb onto the active sites of the HA adsorbent, causing a rapid increase in adsorption capacity (Figure 3). As the contact time progresses, the active sites become occupied by Cu ions, reducing their availability and leading to equilibrium on the Chem-HA and Bio-HA surfaces. The adsorption capacity of Chem-HA was approximately 69.8 mg/g, while the adsorption capacity of Bio-HA at equilibrium was approximately 51.0 mg/g. Chem-HA exhibits higher adsorption capacity than Bio-HA during the 4 h contact time process, which may be attributed to the lower crystallinity of Chem-HA synthesized from calcium oxide. This lower crystallinity likely facilitates easier ion exchange between Cu ions and Ca ions. Guo et al. [37] investigated the adsorption characteristics of nano-HA for Cu ions and found that the maximum adsorption capacity of 60.98 mg/g was achieved at a Cu ion concentration of 350 mg/L.

Table 1 shows the kinetic parameters of Chem-HA and Bio-HA in the adsorption of Cu ions. These parameters include the pseudo-first-order and pseudo-second-order rate constants (k_1_ and k_2_) and their corresponding correlation coefficients (R^2^). For Chem-HA, the pseudo-first-order rate constant k_1_ is 2.41 × 10^−3^ min^−1^, with a correlation coefficient R^2^ of 0.756, indicating a poor fit of this model to the data. In contrast, the pseudo-second-order rate constant k_2_ is 5.16 × 10^−4^ g/mg∙min, with a correlation coefficient R^2^ of 0.989, suggesting that the pseudo-second-order model better describes the Cu ion adsorption process of Chem-HA. Similarly, for Bio-HA, the pseudo-first-order rate constant k_1_ is 1.60 × 10^−3^ min^−1^, with a correlation coefficient R^2^ of 0.779, also indicating a poor fit. However, the pseudo-second-order rate constant k_2_ is 6.39 × 10^−4^ g/mg∙min, with a correlation coefficient R^2^ of 0.998, showing that the pseudo-second-order model better fits the Cu ion adsorption process of Bio-HA. The results of Wang et al. [38] indicate that the adsorption kinetics of Cu ions onto HA are best described by the pseudo-second-order model.

The pseudo-second-order rate constant k_2_ indicates the rate of chemical adsorption, reflecting the interaction between the active sites on the adsorbent surface and the target ions. Higher values of k_2_ indicate a faster and more efficient adsorption process. The k_2_ value for Bio-HA (6.39 × 10^−4^ g/mg·min) is slightly higher than that for Chem-HA (5.16 × 10^−4^ g/mg·min), indicating that Bio-HA has a slightly faster adsorption rate and better adsorption performance under the pseudo-second-order model. The high correlation coefficients (R^2^) of the pseudo-second-order model further confirm this, showing that the adsorption process is primarily controlled by a chemical adsorption mechanism.

### 3.3. Langmuir and Freundlich Isotherm Models

**Figure 4:**
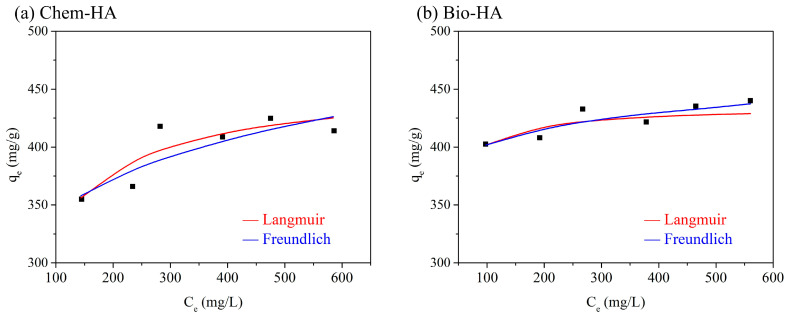
Adsorption isotherms of Cu ions on (**a**) Chem-HA and (**b**) Bio-HA. The figure displays the Langmuir and Freundlich isotherm models fitted to the experimental data of both adsorbents.

Figure 4 shows the adsorption isotherms of Cu ions on Chem-HA and Bio-HA, illustrating the Langmuir and Freundlich isotherm models that best fit the experimental data of both adsorbents. The maximum adsorption capacity (q_m_) of Chem-HA is approximately 426.7 mg/g, while the maximum adsorption capacity of Bio-HA is approximately 436.8 mg/g. Table 2 presents the Langmuir and Freundlich parameters for the adsorption of Cu ions by Chem-HA and Bio-HA. According to the Langmuir model, Chem-HA has a maximum adsorption capacity (q_e_) of 426.6 mg/g, a Langmuir constant (K_L_) of 0.0251 L/mg, and a high correlation coefficient (R^2^) of 0.9961, indicating a good fit to the model. This suggests that the adsorption occurs predominantly on a homogeneous surface with monolayer coverage. In comparison, Bio-HA has a slightly higher maximum adsorption capacity (q_e_) of 428.5 mg/g, a significantly higher Langmuir constant (K_L_) of 0.1229 L/mg, and an even higher correlation coefficient (R^2^) of 0.9989, demonstrating an excellent fit to the Langmuir model and indicating effective monolayer adsorption on a homogeneous surface. The small differences in q_e_ values observed may be attributed to the potentially insufficient volume of the Cu ion solution or the excessive amount of sample added.

In the Freundlich model, Chem-HA has a Freundlich constant (K_F_) of 192.4 (L^1/n^·mg^(1−1/n)^·g^−1^), an adsorption intensity (1/n) of 0.125, and a lower correlation coefficient (R^2^) of 0.7037, suggesting a less satisfactory fit to the model. This indicates that Chem-HA’s surface may not be highly heterogeneous and that multilayer adsorption is less significant. Conversely, Bio-HA has a higher Freundlich constant (K_F_) of 317.9 (L^1/n^·mg^(1−1/n)^·g^−1^), an adsorption intensity (1/n) of 0.0507, and a higher correlation coefficient (R^2^) of 0.8083, showing a better fit to the Freundlich model. This suggests that Bio-HA’s surface is relatively more heterogeneous, with more significant multilayer adsorption.

The higher K_F_ value for Bio-HA suggests that Bio-HA has a higher affinity for Cu ions compared to Chem-HA. This increased capacity could be attributed to the presence of more adsorption sites on Bio-HA, which results from the natural impurities and structural features of the clam shell-derived material. On the other hand, the higher 1/n value for Chem-HA reflects a more heterogeneous adsorption process, implying that Chem-HA has a broader range of adsorption sites with varying affinities for the Cu ions. However, it is noteworthy that the 1/n values for both Chem-HA and Bio-HA are less than 1, indicating that the adsorption sites of both materials are relatively uniform and that the adsorption process is favorable. This suggests that both Chem-HA and Bio-HA exhibit a relatively homogeneous distribution of adsorption sites, which supports efficient metal ion removal.

Overall, the experimental results indicate that the adsorptions of Cu ions by both Chem-HA and Bio-HA are better described by the Langmuir model, highlighting that the adsorption primarily occurs on a homogeneous surface with monolayer coverage. Bio-HA exhibits higher Langmuir and Freundlich constants compared to Chem-HA, indicating superior adsorption capacity and affinity for Cu ions. Additionally, the higher correlation coefficient for Bio-HA in the Freundlich model suggests a more heterogeneous surface, contributing to its better adsorption performance. In summary, Bio-HA shows better performance in Cu ion adsorption under the studied conditions, making it a more effective adsorbent compared to Chem-HA.

Table 3 presents a comparative analysis of the maximum Cu ion adsorption capacities across various HA-based adsorbents. The results from our study demonstrate superior performance, with Chem-HA and Bio-HA achieving q_m_ values of 426.7 mg/g and 436.8 mg/g, respectively. These values significantly surpass those of many other adsorbents reported in the literature, such as synthetic HA, which exhibits a wide range of adsorption capacities (29.2–517.1 mg/g), and natural HA, which generally shows lower capacities (10.6–100.0 mg/g). Notably, the q_m_ of 426.7 mg/g for Chem-HA and 436.8 mg/g for Bio-HA are among the highest reported, highlighting their exceptional efficiency in Cu ion adsorption. This underscores the potential of both Chem-HA and Bio-HA as highly effective adsorbents in environmental applications.

### 3.4. Thermodynamic Analysis

**Figure 5:**
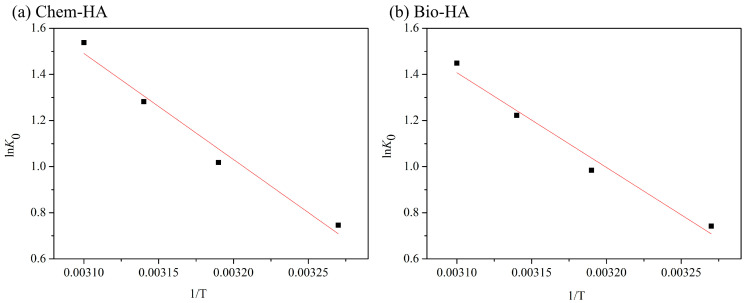
Thermodynamics fitting of Cu ion adsorption by (**a**) Chem-HA and (**b**) Bio-HA powders.

The thermodynamic parameters for the adsorption of Cu ions on Chem-HA and Bio-HA at various temperatures indicate key insights into their adsorption behavior. The thermodynamic experiments were conducted at temperatures ranging from 308 to 323 K, with initial conditions involving the addition of 0.02 g of adsorbent to 20 mL of a 500 mg/L Cu ion solution. Figure 5 illustrates thermodynamic fitting curves of Cu ion adsorption on Chem-HA and Bio-HA. The results indicate that the adsorption capacity of both Chem-HA and Bio-HA increases with rising temperature. Thermodynamic parameters were calculated using the formula ln K_0_ = ∆S°/R − ∆H°/RT, as summarized in Table 4.

At 308 K, Chem-HA has ∆G of –1.91 kJ/mol, ∆H° of 43.77 kJ/mol, and ∆S° of 148.30 J/mol·K. In comparison, Bio-HA at the same temperature has ∆G of –1.90 kJ/mol, ∆H° of 39.00 kJ/mol, and ∆S° of 132.80 J/mol·K. As the temperature increases, the ∆G values for both adsorbents become more negative, indicating that the adsorption process becomes more spontaneous at higher temperatures. Chem-HA has a significantly higher ∆H° compared to Bio-HA, suggesting that the adsorption process of Chem-HA is endothermic and requires more energy, indicating a stronger interaction with Cu ions. In contrast, Bio-HA has a lower ∆H°, indicating that less energy is required for the adsorption process. Regarding ∆S°, Chem-HA shows a higher value than Bio-HA, reflecting a greater increase in disorder during the adsorption process. Overall, Chem-HA exhibits stronger adsorption performance and higher energy demands for Cu ion adsorption, while Bio-HA also demonstrates good adsorption capacity, albeit with lower enthalpy and entropy changes. This suggests that while Chem-HA is more effective under certain conditions due to its stronger interactions, Bio-HA still presents significant adsorption potential with comparatively lower energy requirements and changes in disorder.

Using the method described by Bazargan-Lari et al. [12], the sticking probability (S*) and activation energy (E_a_) for Chem-HA and Bio-HA can be further calculated from the ΔG, ΔH°, and ΔS° values provided in Table 4. S* is a dimensionless parameter that represents the likelihood of an ion being adsorbed upon collision with a solid surface. It is defined as the ratio of successfully adsorbed molecules to the total number of collisions. The E_a_ value was obtained by applying the Arrhenius equation to the rate constant k_2_ derived from the pseudo-second-order kinetic model. The calculated S*s for both Chem-HA and Bio-HA are approximately 0.467. This value indicates a moderate likelihood of Cu ions adhering to the surfaces of both adsorbents, with the adsorption process primarily governed by a physisorption mechanism. Specifically, Chem-HA has E_a_ of 46.39 kJ/mol, while Bio-HA has an activation energy of 41.62 kJ/mol. These values reveal the energy requirements and efficiency of the adsorption processes for Cu ions. Chem-HA’s E_a_ of 46.39 kJ/mol suggests that its adsorption process requires a higher amount of energy, indicating stronger interactions between Chem-HA and Cu ions. This higher activation energy implies that the adsorption process is more temperature sensitive, and Chem-HA’s adsorption efficiency is likely to improve at higher temperatures. Although Chem-HA’s process requires more energy, it indicates a potential advantage in providing stronger adsorption forces, making it suitable for applications needing high temperatures or enhanced adsorption strength. In contrast, Bio-HA’s E_a_ of 41.62 kJ/mol is slightly lower than that of Chem-HA, suggesting that its adsorption process demands less energy and may be simpler. This lower activation energy indicates that Bio-HA can maintain effective adsorption capacity under varying temperature conditions with less energy input. This makes Bio-HA potentially more flexible and adaptable in different operational conditions.

### 3.5. Adsorption Mechanisms

**Figure 6:**
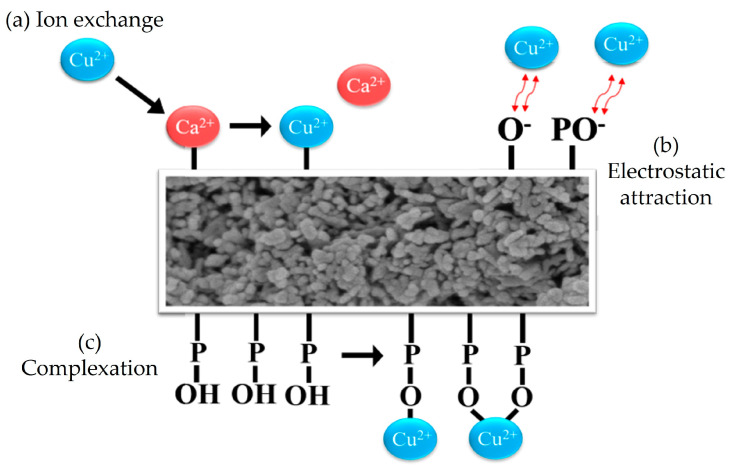
Possible mechanisms of Cu ion adsorption by HA in this study.

Figure 6 illustrates the potential mechanisms of Cu ion adsorption by HA in this study. The three primary mechanisms are ion exchange, electrostatic attraction, and complexation. Ion exchange involves the replacement of Ca ions on the HA surface or within its lattice by Cu ions from the solution. This process occurs because Cu and Ca have similar charges and ionic radii, allowing effective ion exchange and resulting in the fixation of Cu ions on the HA surface. Electrostatic attraction refers to the attraction between positively charged Cu ions in the solution and negatively charged sites on the HA surface. The negative charges on the HA surface arise from the partial dissociation of hydroxyl or phosphate groups. As Cu ions approach the HA surface, they are attracted by these electrostatic forces and become adsorbed onto the surface. Complexation describes the formation of complexes between Cu ions and functional groups on the HA surface, such as phosphate or hydroxyl groups. This process involves the creation of coordination bonds between Cu ions and oxygen atoms from the phosphate or hydroxyl groups in HA. These coordination bonds stabilize the Cu ions on the HA surface, enhancing the adsorption and making it less likely for the Cu ions to be desorbed. When HA is used as an adsorbent for Cu ions in solution, these three mechanisms—ion exchange, electrostatic attraction, and complexation—may occur simultaneously or sequentially, depending on the solution’s pH, ion concentration, surface properties of HA, and other environmental conditions.

## 4. Conclusions

This study investigates Chem-HA and Bio-HA powders, synthesized from chemical calcium oxide and clam shells, respectively, and their Cu ion adsorption capabilities. Both Chem-HA and Bio-HA are identified as HA, with Bio-HA exhibiting higher crystallinity (67.5%) compared to Chem-HA (34.9%). The adsorption of Cu ions follows a pseudo-second-order model for both types. The Langmuir model best describes the adsorption process, indicating a more homogeneous distribution of adsorption sites, though the Freundlich model also fits well, suggesting some site heterogeneity. The maximum adsorption capacities were 436.8 mg/g for Bio-HA and 426.7 mg/g for Chem-HA, as determined by the Langmuir isotherm model. Thermodynamic studies reveal that the adsorption is spontaneous and endothermic, with positive ΔH° and ΔS° values, indicating that higher temperatures enhance Cu-ion uptake. Overall, both Chem-HA and Bio-HA effectively adsorb Cu ions, with Bio-HA demonstrating better energy efficiency and environmental benefits due to its natural origin and recyclability.

## Figures and Tables

**Table 1:** The kinetic parameters of Chem-HA and Bio-HA in the adsorption of Cu ions.

Sample	Pesudo-First-Order Kinetic Model		Pesudo-Second-Order Kinetic Model	
	k_1_ (1/min)	R^2^	k_2_ (g/mg·min)	R^2^
Chem-HA	2.41 × 10^−3^	0.756	5.16 × 10^−4^	0.989
Bio-HA	1.60 × 10^−3^	0.779	6.39 × 10^−4^	0.998

**Table 2:** Langmuir and Freundlich isotherm model parameters for Chem-HA and Bio-HA.

Sample	Langmuir Isotherm Model			Freundlich Isotherm Model		
	q_e_ (mg/g)	K_L_ (L/mg)	R^2^	K_F_ L^1/n^·mg^(1−1/n)^·g^−1^	1/n	R^2^
Chem-HA	426.6	0.0251	0.9961	192.4	0.125	0.7037
Bio-HA	428.5	0.1229	0.9989	317.9	0.0507	0.8083

**Table 3:** Comparison of maximum Cu ion adsorption capacities for various HA adsorbents.

Adsorbent	q_m_ (mg/g)	References
Chem-HA	426.7	This study
Bio-HA	436.8	This study
Synthetic HA	31.6	[39]
Synthetic HA	29.2	[40]
Synthetic HA	517.1	[41]
Synthetic HA	55.9	[42]
Natural HA	100.0	[43]
Natural HA	10.6	[24]
Nano-HA/chitosan biocomposites	237.0	[44]
HA/biochar nanocomposites	99.0	[45]
HA/geopolymer	13.7	[46]
HA/cellulose-g-poly(acrylamide)	175.0	[47]
HA-bound Fe_3_O_4_ magnetic nanoparticles	48.8	[48]

**Table 4:** Thermodynamic parameters for the adsorption of Cu ions on Chem-HA and Bio-HA at various temperatures.

T (K)	∆G (kJ/mol)	∆H° (kJ/mol)	∆S° (J/mol·K)
Chem-HA			
308	–1.91	43.77	148.30
313	–2.65		
318	–3.39		
323	–4.13		
Bio-HA			
308	–1.90	39.00	132.80
313	–2.56		
318	–3.23		
323	–3.89		

## Data Availability

The data are contained within the article.
